# Subjective cognitive decline, and its cognitive and emotional correlates in Peruvian older adults

**DOI:** 10.1590/1980-5764-DN-2025-0419

**Published:** 2026-04-20

**Authors:** Marcio Soto-Añari, Claudia Rivera-Fernández, Manuel Montemurro, Francisco Ceric, María del Carmen Tejada, Andrés Muñoz-Najar, María Gracia Carbajal, Edmarie Guzmán-Vélez, Yakeel T. Quiroz

**Affiliations:** 1 Universidad Católica San Pablo, Laboratorio de Neurociencia Cognitiva, Arequipa, Perú.; 2 Universidad Tecnológica del Perú, Arequipa, Perú.; 3 Universidad del Desarrollo, Instituto de Bienestar Socioemocional, Santiago, Chile.; 4 Universidad del Alba, Escuela de Psicología, Santiago, Chile.; 5 Harvard Medical School, Massachusetts General Hospital, Department of Psychiatry, Boston MA, United States of America.; 6 Boston University, Department of Psychological & Brain Sciences, Boston MA, United States of America.

**Keywords:** Cognitive Dysfunction, Neuropsychological Tests, Emotions, Aging, Depression, Disfunción Cognitiva, Pruebas Neuropsicológicas, Emociones, Envejecimiento, Depresión

## Abstract

Subjective cognitive decline (SCD) is a known risk factor for dementia, yet its association with objective cognition and emotional state remains underexplored in Latin American populations.

**Objective:**

The aim of this study was to explore the relationship between SCD, objective cognition, and emotional state in older adults from Arequipa, Peru.

**Methods:**

This study investigated these relationships in 43 older adults from Arequipa, Peru, classified as having SCD (n=17), mild cognitive impairment (n=12), or normal cognition (n=14). Participants underwent assessments of global cognition, subjective decline (via the E-Cog), objective cognitive domains, and emotional state.

**Results:**

Findings showed a significant association between SCD and emotional disorders, with emotional symptoms correlating with total E-Cog scores and specific domains such as memory, language, planning, and organization. Additionally, general cognition, long-term memory, and cognitive flexibility were associated with deficits in visuospatial processing, attention, and executive function.

**Conclusions:**

These results underscore the need to account for emotional factors when evaluating the relationship between SCD and objective cognitive performance.

## RESUMEN.

El declive cognitivo subjetivo (DCS) es un factor de riesgo reconocido para la demencia; sin embargo, su asociación con la cognición objetiva y el estado emocional permanece poco explorada en poblaciones latinoamericanas.

**Objetivo:**

Conocer la relación entre el DCS, la cognición y el estado emocional en adultos mayores de Arequipa, Perú.

**Métodos:**

Este estudio investigó dichas relaciones en 43 adultos mayores de Arequipa, Perú, clasificados con DCS (n=17), deterioro cognitivo leve (n=12) o cognición normal (n=14). Los participantes fueron evaluados en cognición global, declive subjetivo (mediante el E-Cog), dominios cognitivos objetivos y estado emocional.

**Resultados:**

Los hallazgos mostraron una asociación significativa entre DCS y trastornos emocionales, observándose que los síntomas emocionales se correlacionaron con las puntuaciones totales del E-Cog y con dominios específicos como memoria, lenguaje, planificación y organización. Asimismo, la cognición general, la memoria a largo plazo y la flexibilidad cognitiva se asociaron con déficits en el procesamiento visuoespacial, la atención y la función ejecutiva.

**Conclusiones:**

Estos resultados subrayan la necesidad de considerar los factores emocionales al evaluar la relación entre DCS y el rendimiento cognitivo objetivo.

## INTRODUCTION

 Older adults often report difficulties with cognitive functions like memory^1^, even when standardized cognitive assessments show no significant impairment, a phenomenon known as subjective cognitive decline (SCD)^2^. SCD affects 50–80% of older adults^3^ and is considered an early marker of Alzheimer’s disease^4^ and increased dementia risk^5,6^. 

 Approximately 27% of SCD cases progress to mild cognitive impairment (MCI), and 14% to dementia^7^, with higher prevalence among women and those with mental health issues^8^. Depressive symptoms and emotional distress may amplify SCD and accelerate cognitive decline^9-11^, although some evidence suggests SCD can be linked to objective changes even in the absence of emotional symptoms^12^. 

 Most findings on the interplay between SCD, emotional distress, and objective cognitive performance^13^, primarily come from North American and European samples^14-15^, with limited evidence in Latin American populations. 

 Some studies in U.S. Latino groups found associations between SCD and general cognition^16^, while others reported no link between SCD and objective measurements, either with general cognition^17^ or specific cognitive dimensions^18^. Additionally, it has been observed that older Hispanic populations with depressive symptoms report a greater number of SCD cases compared to non-Hispanic populations. Notably, Hispanic older adults with depressive symptoms report higher SCD rates than non-Hispanic peers, yet little is known about these dynamics outside the United States^19^. 

 The COVID-19 pandemic exacerbated emotional distress^20^, potentially heightening both SCD and objective cognitive decline. However, no studies have explored how emotional factors may shape the relationship between subjective complaints and cognitive performance in older adults in Peru. This study addresses this gap by examining the relationship between SCD, objective cognition, and emotional state in older adults from Arequipa, Peru. 

## METHODS

### Participants

 Forty-three adults aged 55–86 years (*M*=69.72, *SD*=6.80) were recruited from public senior centers in Arequipa, Peru (Table 1). Participants were excluded if they had a diagnosis of dementia, neurological disease or neuropsychiatric disorder; a history of alcohol or drug abuse; severe sensory or perceptual deficits; a Pfeffer Functional Questionnaire score >6; or a clinical dementia Rating (CDR) score >0.5. 

**Table 1:** Demographic and clinical variables.

		Groups			
		CH (n=14)^1^	SCD (n=17)^1^	MCI (n=12)^1^	p-value
Age		68.36 (6.25)	70.18 (6.38)	70.67 (8.21)	0.658
Education in years		15.36 (3.41)	13.65 (4.13)	11.92 (5.40)	0.141
Sex	Male	2 (14.3)	2 (11.8)	1 (8.3)	0.894
	Female	12 (85.7)	15 (88.2)	11 (91.7)	
Mariage status	Single	1 (7.1)	0 (0)	0 (0)	0.828
	Married	7 (50)	12 (70.6)	9 (75)	
	Divorced	1 (7.1)	1 (5.9)	0 (0)	
	Separated	2 (14.3)	1 (5.9)	1 (8.3)	
	Widower	3 (21.4)	3 (17.6)	2 (16.7)	
Occupation	Professional	6 (42.9)	5 (29.4)	3 (25)	0.752
	Technician	3 (21.4)	5 (29.4)	2 (16.7)	
	Trader	3 (21.4)	2 (11.8)	2 (16.7)	
	Housewife	2 (14.3)	5 (29.4)	5 (41.7)	
Diabetes	No	12 (85.7)	15 (88.2)	8 (66.7)	0.299
	Yes	2 (14.3)	2 (11.8)	4 (33.3)	
Cardiovascular disease	No	11 (78.6)	8 (47.1)	8 (66.7)	0.185
	Yes	3 (21.4)	9 (52.9)	4 (33.3)	

Abbreviations: CH, Cognitively healthy; SCD, Subjective cognitive decline; MCI, Mild cognitive impairment.

Note: ^1^mean/n (SD/%); No statistically significant differences were observed between SCD, MCI and Cognitively Healthy (CH) groups for all variables.

 The evaluations were conducted in two phases. In the first phase, assessments of emotional status and SCD were administered by phone during the COVID-19 pandemic. In the second phase, participants were invited to complete an extensive neuropsychological protocol in person. The interval between the two phases was less than 6 months. In the first phase, 14 participants did not have SCD or cognitive impairment (i.e., cognitively healthy [CH]), 17 were classified with SCD according to the criteria of Molinuevo et al.^4^, and 12 met the criteria for MCI^21^. In the second phase, only 32 participants (CH=10, SCD=14, and MCI=8) completed the extensive neuropsychological protocol. Eleven participants dropped out: eight declined to continue, and three were excluded after meeting the criteria for mild dementia. The participants in the healthy control and SCD groups both exhibited Mini-Mental State Examination (MMSE) score>27, showed performance above the 25th percentile on the neuropsychological assessment and had a Geriatric Depression Scale (GDS) score of 0. Additionally, SCD participants reported a subjective decline in cognitive capacity compared with their previous state. For the MCI group, the diagnosis included MMSE score<27, GSD score=0.5, and performance below the 25th percentile in one or more cognitive domains, according to Peruvian normative data. 

### Instruments

#### Objective measurement

 Participants were classified in three groups (CH, SCD, and MCI) based on the MMSE, a measure of global cognition, the Pfeffer Functional Activities Questionnaire, and the CDR. Memory was assessed using the Free and Cued Selective Reminding Test (FCSRT) and Rey-Osterrieth Complex Figure recall; attention and executive function with the Trail Making Test (TMT-A and B), the Stroop test, and the modified version of the Wisconsin Card Sorting Test (WCST-M); working memory with forward and backward digit span from the Wechsler Adult Intelligence Scale; verbal fluency with phonological tasks (letters F, A, and S), and semantic (animals and fruits) tasks; and visuospatial function with the Rey-Osterrieth Complex Figure copy. For details on the clinical and neuropsychological protocol, see Rivera-Fernández et al.^22^. 

#### Subjective cognitive decline assessment

 The Everyday Cognition questionnaire (E-Cog) was used to measure SCD (For more details on the criteria, instruments, and procedures, see Babulal et al.^23^; Farias et al.^24^). The E-Cog questionnaire consists of 39 items divided into four cognitive domains: memory, language, visuospatial, and perceptual abilities, as well as executive functioning, including planning, organization, and attention^24^. 

#### Emotional assessment

 For the emotional distress assessment, the Beck’s Depression Inventory II (BDI-II)^25^ and the emotional impact subscale of the Epidemic-Pandemic Impacts Inventory (EPII)^26^ were used. The EPII’s emotional subscale is an eight-item scale which assesses the impact of the COVID-19 or other public health issues on emotional health. 

### Statistical analyses

 Spearman’s rho and Spearman partial correlations were used to examine associations between neuropsychological measures, controlling for emotional variables, due to their impact on subjective cognition. Non-parametric tests were applied due to the non-normal sample distribution. Statistical analyses were conducted in *Jamovi* v.2.3.21, with a significance threshold of 0.05. 

### Ethics considerations

 This study followed the guidelines of the Declaration of Helsinki and was approved by the ethics committee of the Research Directorate of the Universidad Católica San Pablo according to Act No. 001.CEDI.UCSP.2019. All participants provided written informed consent before participating in the study. 

## RESULTS

 Significant group differences (CH, SCD, and MCI) were found across several measures, including the MMSE (*F*_2_, _40_=34.89, p<0.001, *η* ^2^=0.64), E-Cog language (*F*_2_, _40_=3.76, p=0.032, *η* ^2^=0.16), E-Cog visuospatial (*F*_2_, _40_=7.27, p=0.002, *η* ^2^=0.27), E-Cog total score (*F*_2_, _40_=4.70, p=0.015, *η* ^2^=0.19), as well as visual memory (*F*_2_, _29_=3.85, p=0.033, *η* ^2^=0.21), digits backward (*F*_2_, _29_=5.85, p=0.007, *η* ^2^=0.29), phonological fluency—F (*F*_2_, _29_=4.23, p=0.024, *η* ^2^=0.23), semantic fluency—animals (*F*_2_, _29_=8.02, p=0.002, *η* ^2^=0.36), semantic fluency—fruits (*F*_2_, _29_=4.35, p=0.022, *η* ^2^=0.23), and visuospatial processing (*F*_2_, _29_=4.75, p=0.016, *η* ^2^=0.25). For detailed post hoc results, see Supplementary Material 1. 

 BDI-II scores were significantly correlated with E-Cog total scores (*rho*=0.35, p<0.05), and with subscales for memory (*rho*=0.43, p<0.01), language (*rho*=0.45, p<0.01), planning (*rho*=0.31, p<0.05) and organization (*rho*=0.31, p<0.05) (see Supplementary Material 2). Additionally, the EPII emotional subtest was associated with the E-Cog total score (*rho*=0.34, p<0.05) and the memory subscale (*rho*=0.38, p<0.05). A negative correlation was found between MMSE and the visuospatial subscale of the E-Cog (*rho*=−0.46, p<0.01). The E-Cog planning subscale was negatively associated with the long-term free recall of the FCSRT (*rho*=-0.505, p<0.01). Similarly, the E-Cog attention subscale was significantly related to the number of categories of the WCST (*rho*=0.387, p<0.05). No other significant relationships were observed between objective neuropsychological measurements and the E-Cog. 

 Emotional variables (BDI-II and EPII-emotional scale) were controlled in the analysis of associations between E-Cog scores and objective measures across the entire sample. Only significant partial correlations are presented in Table 2. 

**Table 2:** Correlation matrix among objective and subjective measurements controlled by emotional distress.

	E-Cog memory	E-Cog visuospatial	E-Cog planification	E-Cog attention	E-Cog total
MMSE	—	-0.460** ^a^	—	—	-0.350* ^b^
	—	-0.470** ^b^	—	—	
	—	-0.530*** ^c^	—	—	-0.400** ^c^
	-0.310* ^d^	-0.530*** ^d^	—	—	-0.410** ^d^
Visual memory—Rey M1	—	-0.403* ^c^	—	—	-0.417* ^c^
	—	-0.400* ^d^	—	—	-0.416* ^d^
STROOP PC	—	—	-0.365* ^c^	—	—
	—	—	-0.363* ^d^	—	—
Long-term memory—second trial	—	—	—	—	0.363* ^d^
Long-term memory—free recall	—	—	-0.505** ^a^	—	—
	—	—	-0.483** ^b^	—	—
	—	—	-0.546** ^c^	—	—
	—	—	-0.522** ^d^	—	—
WCST—Cat	—	—	0.387* ^a^	—	—
TMT—A Err	—	-0.350* ^a^	—	—	—
	—	-0.364* ^b^	—	—	—
TMT—B Err	—	—	—	-0.379* ^a^	—
	—	—	—	-0.403* ^c^	—
Digit backward	—	—	-0.371* ^c^	—	—
Phonological and semantic lexical access—Animals	—	—	-0.365* ^c^	—	—

Notes: ^a^ Bivariate correlation, ^b^ Controlled by BDI-II, ^c^ Controlled by EPI-II, ^d^ Controlled by BDI-II and EPI-II. *p<0.05, **p<0.01, ***p<0.001.

 MMSE scores showed negative correlations with E-Cog visuospatial and total scores across all control conditions. The Rey-M1 scores showed negative correlations with E-Cog visuospatial and total scores in two control conditions. Stroop-PC’ showed negative correlations with E-Cog planification in two control conditions. Long-term memory—second trial, showed positive correlation with E-Cog total controlled by both emotional measurements. Long-term memory—free recall showed negative correlations with E-Cog planification across all control conditions. WCST—categories showed positive bivariate correlation with E-Cog planification. TMT-A errors showed negative correlations with E-Cog visuospatial in two control conditions, and TMT-B errors negative correlations with E-Cog attention in two control conditions. Digit backward and phonological and semantic lexical access—Animal, both showed negative correlations with E-Cog planification controlled by EPI-II. 

## DISCUSSION

 This study examined the relationship between SCD, objective cognition, and emotional distress in older adults in Peru. Significant associations emerged between depressive symptoms and subjective cognitive decline, particularly in memory, language, and planning, suggesting that emotional distress may influence the perception of cognitive decline. 

 Emotional distress during the COVID-19 pandemic was also linked to higher E-Cog total and memory subscale scores, consistent with the idea that affective symptoms may influence cognitive self-assessment, and consistent with previous reports^18^. Notably, lower MMSE scores correlated with increased visuospatial complaints, consistent with prior findings that visuospatial deficits may signal early cognitive decline^22,27^. Participants with MCI reported greater difficulty with everyday spatial tasks, such as navigation and orientation. 

 The study also found that poor long-term recall was associated with higher planning complaints, possibly reflecting reduced use of strategic memory processes. Similarly, attentional complaints correlated with performance on the WCST, suggesting that perceived cognitive lapses may reflect difficulty sustaining attention or filtering distractions, patterns documented in both normal^28^ and pathological aging^29^. 

 These findings raise the possibility that inconsistencies between subjective complaints and objective performance may be partly explained by unaccounted emotional variables. Controlling for emotional distress, especially during the pandemic, suggested stronger links between subjective and objective cognition, including new associations between short-term memory and global complaints, and between planning difficulties and inhibitory control (Stroop test). 

 It is worth noting that depressive symptoms and pandemic-related emotional distress did not differ significantly across cognitive groups yet were included as covariates due to their potential influence on subjective perception and cognitive functioning at the individual level. While overall levels of emotional symptoms did not differ significantly across cognitive groups, our findings suggest that distinct emotional dimensions may be differentially associated with cognitive performance and self-perception. Internal affective states, such as persistent low mood or reduced interest, have been consistently linked to impairments in attention, processing speed, and working memory^30^. In contrast, emotionally salient contextual stressors—such as disrupted routines, isolation, or uncertainty—may compromise executive functioning and amplify subjective perceptions of cognitive decline^31^. Studies conducted during the COVID-19 pandemic have shown that both affective symptoms and environmental distress independently predict increased cognitive complaints and reduced memory and attentional performance, particularly among older adults^32^. These distinctions underscore the importance of considering both internal and situational dimensions of emotional distress when interpreting the relationship between subjective and objective cognition. Given the exploratory nature of this study and the modest sample size, these associations should be interpreted with caution. 

 While promising, results are limited by the small sample size, which constrains generalizability and the ability to predict dementia risk. Future studies should include larger and diverse samples, distinguish between subtypes of MCI, incorporate biomarkers for diagnostic validation and reduce gender imbalance. Pandemic-related stress may also have influenced participants’ cognitive and emotional functioning^20^. 

 Strengths of this study include the detailed analysis of subjective complaints, particularly in language, which emerged as a potential early indicator of cognitive decline, and the systematic consideration of emotional variables. Together, these approaches enhanced the validity of findings and underscored the importance of including emotional measures when examining subjective-objective relationships. 

## Data Availability

The datasets generated and/or analyzed during the current study are available from the corresponding author upon reasonable request.
