# Supplementary material for: Subjective cognitive decline, and its cognitive and emotional correlates in Peruvian older adults
Source: Dement Neuropsychol. 2026 Apr 20;20:e2025419. doi: 10.1590/1980-5764-DN-2025-0419 (PMC13105449; doi:10.1590/1980-5764-DN-2025-0419)
Supplement: Supplementary Material 1 [file 1980-5764-dn-20-e2025419-md01.docx]

**Supplementary Material**

**Table S1.** Clinical and Neuropsychological data

| Clinical data | Groups | | | *p* | *post hoc* |
| --- | --- | --- | --- | --- | --- |
|  | *CH*  *(n = 14)*^1^ | *SCD*  *(n = 17)*^1^ | *MCI*  *(n = 12)*^1^ |  |  |
|  |  |  |  |  |  |
| MMSE | 29(1.10) | 28.94(0.75) | 22.83(3.83) | < .001 | CH > MCI ***  SCD > MCI *** |
| Beck Depression Inventory II | 4.43(2.79) | 7.35(4.38) | 6.17(3.04) | .089 |  |
| E-Cog | | | | | |
| *Memory* | 13.86(4.27) | 16.76(4.23) | 18.33(6.71) | .081 |  |
| *Language* | 12.86(3.20) | 16.47(4.58) | 17.83(6.57) | .032 | CH < SCD * |
| *Visuospatial* | 16.50(8.64) | 13.24(4.79) | 25.25(11.79) | .002 | CH < MCI *  SCD < MCI ** |
| *Planification* | 6(1.44) | 7.12(2.11) | 8.25(3.41) | .067 |  |
| *Organization* | 10.07(5.16) | 9.88(4.71) | 13.25(6.67) | .221 |  |
| *Attention* | 6.79(2.99) | 7.29(2.49) | 6.92(2.46) | .858 |  |
| *Total score* | 66.07(13.88) | 70.76(17.05) | 89.83(30.31) | .015 | CH < MCI * |
| Loneliness scale | 11.86(2.79) | 10.18(2.06) | 11.67(2.30) | .113 |  |
| Emotional health subscale - EPII | 2.43(1.45) | 2.65(1.93) | 2.08(1.78) | .696 |  |
| Neuropsychological Assessment | *n = 10* | *n = 14* | *n = 8* |  |  |
| Verbal Memory | | | | | |
| *Total recall* | 36.10(3.31) | 31.50(5.47) | 33.50(7.92) | .164 |  |
| *Long term Free recall* | 13.90(1.37) | 13.21(1.92) | 12.25(4.26) | .413 |  |
| Visual memory - Rey M1 | 19.70(9.10) | 15.10(5.34) | 11.06(4.61) | .033 | CH > SCD * |
| Attention - Executive function | | | | | |
| *WCST – Cat.* | 4.40(1.89) | 4.64(1.27) | 3.88(1.64) | .554 |  |
| *WCST - Ep* | 5.10(3.17) | 4.71(5.64) | 5.38(3.58) | .944 |  |
| *TMT - A* | 60.40(31.43) | 88.50(31.77) | 110.63(115.51) | .253 |  |
| *TMT - B* | 189.10(143.92) | 169.86(58.76) | 185.13(104.40) | .705 |  |
| *Stroop PC* | 33.03(6.47) | 31.32(6.79) | 31.05(6.35) | .772 |  |
| Working memory | | | | | |
| *Digit forward* | 8.60(2.67) | 6.43(1.45) | 7.13(2.32) | .057 |  |
| *Digit backward* | 6(2.70) | 4.14(1.09) | 3.25(1.16) | .007 | CH > MCI ** |
| Phonological and semantic lexical access | | | | | |
| *Letter F* | 12.80(2.97) | 9.29(2.61) | 11.25(3.45) | .024 | CH > SCD * |
| *Letter A* | 13.60(3.62) | 10.14(3.61) | 10.88(2.58) | .058 |  |
| *Letter S* | 13.50(5.56) | 9.50(3.50) | 10(3.16) | .071 |  |
| *Animals* | 21.10(3.44) | 16.36(2.84) | 17.13(2.41) | .002 | CH > SCD **  CH > MCI * |
| *Fruits* | 17.80(3.61) | 14.79(2.86) | 13.88(2.58) | .022 | CH > MCI * |
| Visuospatial - Rey copy | 34.40(1.71) | 30.39(5.73) | 26.31(7.91) | .016 | CH > MCI * |

*Note*. CH: Cognitively healthy, SCD: Subjective cognitive decline, MCI: Mild cognitive impairment, ^1^ mean (SD). * *p* < .05, ** *p* < .01, *** *p* < .001

In the clinical measures, it is observed that the CH group differed from the MCI group (*t*_40_ = 7.26, *p* < .001, *d* = 2.86) and the SCD differed from the MCI group (*t*_40_ = 7.50, *p* < .001, *d* = 2.83). Additionally, the CH group had lower scores in E-Cog language (*t*_40_ = -4.98, *p* = .039, *d* = -1.02) and the E-Cog visuospatial (*t*_40_ = -2.63, *p* = .037, *d* = -1.03) than the MCI group, while the MCI group had higher scores in the E-Cog visuospatial than the SCD group (*t*_40_ = -3.76, *p* = .002, *d* = -1.42). Similarly, it was observed that the CH group had lower total E-Cog scores than the MCI group (*t*_40_ = -2.91, *p* = .018, *d* = -1.14).

Further, the CH group had higher scores than the MCI group in visual memory (*t*_29_ = 8.64, *p* = .030, *d* = 1.31), backward digits (*t*_29_ = 3.27, *p* = .008, *d* = 1.55), lexical-phonological fluency - animals (*t*_29_ = 2.84, *p* = .025, *d* = 1.35), lexical-phonological fluency - fruits (*t*_29_ = 2.71, *p* = .034, *d* = 1.28), and visuospatial processing (*t*_29_ = 3.07, *p* = .014). Similarly, the CH group had higher scores than the SCD group in lexical-phonological fluency - letter F (*t*_29_ = 2.88, *p* = .022, *d* = 1.19) and lexical-phonological fluency - animals (*t*_29_ = 3.88, *p* = .002, *d* = 1.61).

**Table S2.** Correlation matrix among BDI-II, MMSE, and E-Cog measurements

|  | BDI-II | | Emotional health subscale - EPII | | MMSE | |
| --- | --- | --- | --- | --- | --- | --- |
| E-Cog memory | .43 | ** | .38 | * | -.20 |  |
| E-Cog language | .45 | ** | .23 |  | -.11 |  |
| E-Cog visuospatial | .07 |  | .22 |  | -.46 | ** |
| E-Cog planification | .31 | * | .20 |  | -.16 |  |
| E-Cog organization | .31 | * | .07 |  | -.15 |  |
| E-Cog attention | .27 |  | .30 |  | -.05 |  |
| E-Cog total | .35 | * | .34 | * | -.30 |  |
| *Note*. * *p* < .05, ** *p* < .01, *** *p* < .001 | | | | | | |

Spearman’s rank-order correlations revealed moderate, statistically significant positive associations between BDI-II scores and multiple E-Cog domains, including memory (*rho* = .43, *p* < .01), language (*rho* = .45, *p* < .01), planning (*rho* = .31, *p* < .05), organization (*rho* = .41, *p* < .05), and the total E-Cog score (*rho* = .35, *p* < .05). Additionally, the EPII-emotional subscale demonstrated moderate positive correlations with the E-Cog memory domain (*rho* = .38, *p* < .05) and total score (*rho* = .34, *p* < .05). In contrast, MMSE scores showed a moderate and statistically significant negative correlation with the E-Cog visuospatial domain (*rho* = −.46, *p* < .01).
